# Does ginsenoside Rg1 promote intervertebral disc repair? An experimental study insights into ferroptosis mechanism

**DOI:** 10.1186/s12967-025-07047-4

**Published:** 2025-11-06

**Authors:** Dongliang Gong, Feiyun Xia, Fuyong Wang, Xiaoxia Tong, Qing Yang, Kelv Shen, Baihan Sun, Nong Chen, Zhengfeng Lu

**Affiliations:** 1 Department of Orthopedics, The Second Affiliated Hospital of Soochow University, No. 1055 Sanxiang Road, Suzhou, 215004 China; 2 Department of Orthopedics, Qingpu Branch, Zhongshan Hospital Affiliated to Fudan University, Shanghai, 201700 China; 3 Experimental Research Center, Qingpu Branch, Zhongshan Hospital, Fudan University, Shanghai, 201700 China; 4 Department of Orthopedics, Yueyang Integrated Traditional Chinese and Western Medicine Hospital, Shanghai University of Traditional Chinese Medicine, Shanghai, 200437 China

**Keywords:** Ginsenoside Rg1, Intervertebral disc degeneration, Network pharmacology, Transcriptomics, Ferroptosis, NRF2/GPX4 pathway

## Abstract

**Background:**

Intervertebral disc degeneration (IVDD) is a complex and multifactorial condition characterized by the progressive deterioration of the intervertebral discs. Ginsenoside Rg1, a bioactive compound isolated from Panax ginseng C.A.Mey., that has demonstrated promising therapeutic potential in the treatment of IVDD.

**Methods:**

This study employed a multi-faceted approach to investigate the therapeutic effects of ginsenoside Rg1 on IVDD. Initially, histopathology, magnetic resonance imaging (MRI) were performed in clinical IVDD patients. Subsequently, histopathology, safranin green staining, X-ray, and MRI were utilized to evaluate the efficacy of ginsenoside Rg1 in alleviating in a rat model in vivo. Transcriptomics and gene set enrichment analysis (GESA) were conducted, and a network pharmacology visualization of ginsenoside Rg1-ferroptosis key targets-pathways-IVDD was constructed, along with molecular docking of ginsenoside Rg1 and targets, to identify the signaling pathways and proteins associated with the therapeutic effects of ginsenoside Rg1 on alleviating IVDD. Additionally, an Hydrogen peroxide (H_2_O_2_)-induced degeneration model of nucleus pulposus cells (NP cells) was used to evaluate the efficacy of ginsenoside Rg1 in alleviating IVDD *in vitro.* Methods including lipid-reactive oxygen species (ROS) detection, enzyme-linked immunosorbent assay (ELISA), FerroOrange staining, and transmission electron microscopy were employed to validate the effect and mechanism of ginsenoside Rg1 on alleviating IVDD in vivo and in vitro. ML385, a nuclear factor erythroid 2-related factor 2 (NRF2) inhibitor, was used to reverse the effect of ginsenoside Rg1 in mitophagy and ferroptosis, respectively. The expression of proteins was assessed on immunochemical, immunofluorescence, and western blotting techniques.

**Results:**

Significant ferroptosis was observed in the NP tissue of IVDD patients, with more effects in patients with higher imaging grades. Ginsenoside Rg1 significantly mitigated IVDD in rats and promoted intervertebral disc repair. Network pharmacology and transcriptomics analyses indicated the key targets of ginsenoside Rg1 for the treatment of IVDD, including NRF2, glutathione peroxidase 4 (GPX4), solute carrier family 7a member 11 (SLC7A11), and ferritin light chain 1 (FTL1). Molecular docking exhibited that ginsenoside Rg1 had good binding ability between ginsenoside Rg1 and these ferroptosis key targets. Ginsenoside Rg1 reduced the expression of ROS and malondialdehyde (MDA), decreased Fe^2+^ content, increased the expression of glutathione peroxidase (GSH-Px) and superoxide dismutase (SOD), and upregulated the expression of ferroptosis key proteins NRF2, GPX4, FTL1, and SLC7A11 in intervertebral disc tissues and NP cells. Treatment with ML385 attenuated the ginsenoside Rg1-induced upregulation of these proteins in NP cells, thereby promoting ferroptosis and reversing the protective effects of ginsenoside Rg1.

**Conclusions:**

Ginsenoside Rg1 can mitigate IVDD by inhibiting ferroptosis in NP cells. The NRF2/GPX4 pathway was validated as the key ferroptosis pathway through which ginsenoside Rg1 exerts its therapeutic effects on IVDD.

**Graphical Abstract:**

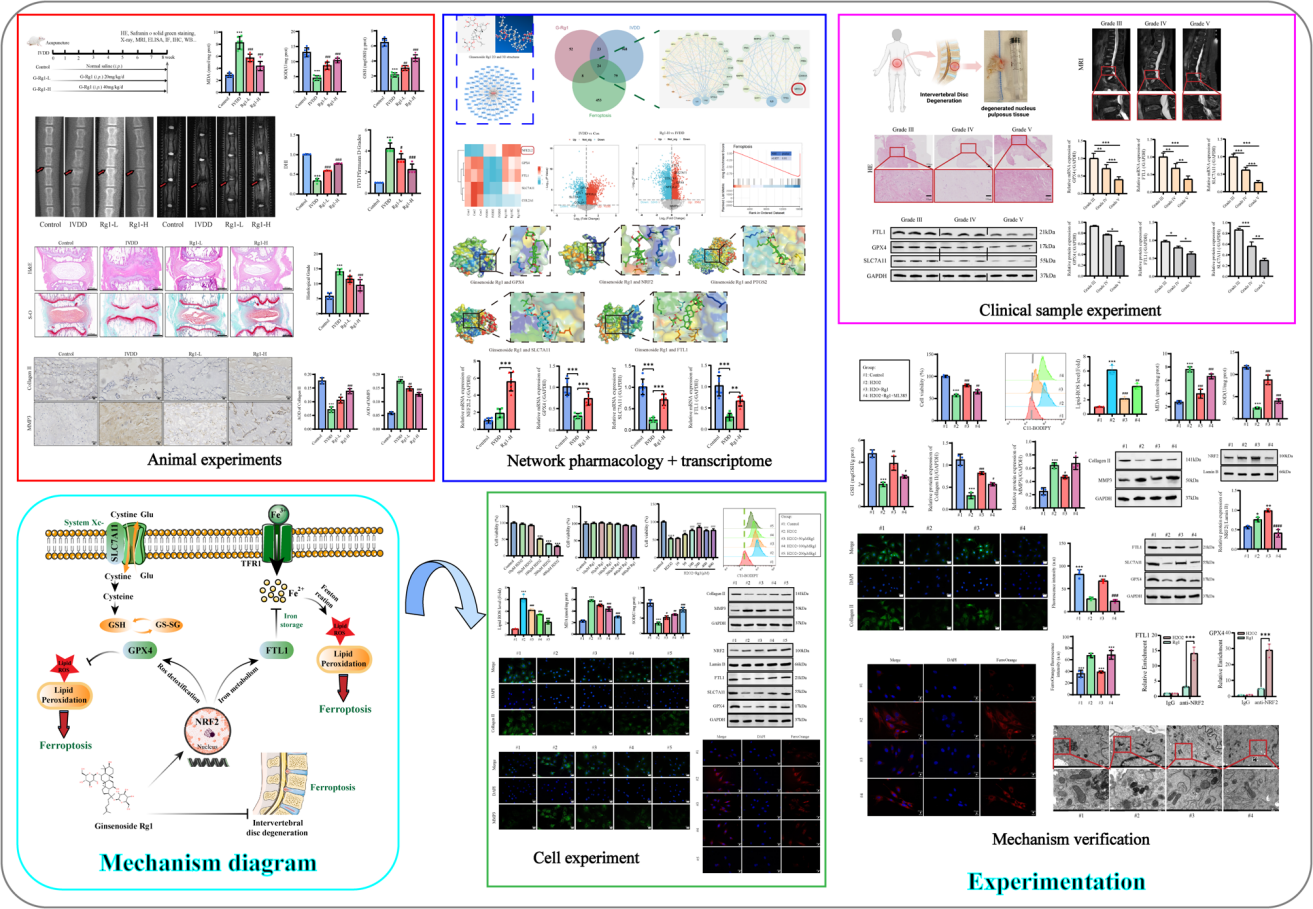

**Electronic supplementary material:**

The online version of this article (doi:10.1186/s12967-025-07047-4) contains supplementary material, which is available to authorized users.

## Introduction

Intervertebral disc degeneration (IVDD) is a pathological process that leads to the deterioration of intervertebral discs [1]. As the primary cause of lower back pain and neuralgia, IVDD affects over 500 million people worldwide, posing a significant burden on public health and the global economy due to its high recurrence and disability rates [2, 3]. The intervertebral disc, a connective tissue between vertebrae, comprises three components: the nucleus pulposus (NP), annulus fibrosus, and cartilage endplates located both above and below the disc [4]. IVDD primarily results from an imbalance between the catabolism and anabolism of the extracellular matrix (ECM), and alterations in the intervertebral disc’s microenvironment [5]. NP cells maintain ECM stability, which is essential for preserving intervertebral disc health [6]. Intervertebral disc cells secrete large amounts of pro-inflammatory factors, triggering pathological reactions that lead to autophagy, aging, and apoptosis, resulting in decreased synthesis of Collagen II and increased expression of matrix metalloproteinases (MMPs) [7]. Ferroptosis, characterized by lipid peroxidation and the Fenton reaction mediated by free iron plays a crucial role in IVDD [8]. Oxidative stress and NP cells death are critical factors in the progression of IVDD [9]. Current clinical treatments for IVDD primarily focus on pain relief and nerve health support, lacking effective drugs to reverse the underlying pathophysiological processes [10].

Regulated cell death, such as apoptosis, necrosis, and pyroptosis, plays a pivotal role in the pathogenesis of degenerative diseases [11]. Ferroptosis, an iron-dependent form of programmed cell death, is distinct from apoptosis and autophagy [12]. Recently, the role of ferroptosis in IVDD has garnered increasing attention from researchers and has been identified as a critical factor in the death of NP cells [13]. The primary mechanism of ferroptosis involves the catalysis of highly expressed unsaturated fatty acids on the cell membrane by divalent iron or ester oxygenase, leading to lipid peroxidation and subsequent cell death [14]. Extensive research indicates that ferroptosis results from the accumulation of ROS due to disrupted iron-dependent Fenton reactions, leading to membrane peroxidation and severe oxidative damage [8, 15, 16].

Ginsenoside Rg1 is the primary active component of Panax ginseng C.A.Mey. Modern medicine acknowledges the extensive pharmacological effects of Panax ginseng CA Mey. On various systems, including the central nervous, cardiovascular, digestive, and immune systems [17]. Although previous studies have demonstrated that ginsenoside Rg1 can suppress the apoptosis of NP cells and promote the synthesis of ECM, which is beneficial for the treatment of IVDD, the precise mechanism remains elusive [18, 19]. Research has indicated that ginsenoside Rg1 enhances the enzymatic antioxidant system and reduces oxidative stress [20]. Furthermore, ginsenoside Rg1 can inhibit the ferroptosis of neurons to ameliorate cognitive impairment and inhibit ferroptosis of renal tubular epithelial cells to ameliorate acute kidney injury [21, 22]. However, to date, there are no published studies investigating the regulation of IVDD by ginsenoside Rg1 in the context of ferroptosis.

This study investigates a potential treatment approach for IVDD by modulating ferroptosis in NP cells. The research employs network pharmacology and transcriptomics to identify key genes and subsequently evaluates the effects and underlying mechanisms of ginsenoside Rg1 on IVDD in rat NP cells through in vitro and in vivo experiments. This objective is to provide a novel therapeutic for the management of IVDD.

## Materials and methods

### Main reagents

Ginsenoside Rg1 (Selleck, S3923, Purity: 99.18%), CCK8 kit (SAB, CP002), Reactive oxygen detection kit (Beyotime, S0033), DCFH-DA (Beyotime, China), FerroOrange (Dojindo, Japan), Malondialdehyde (MDA), Superoxide Dismutase (SOD), Glutathione Peroxidase (GSH-Px) assay kit, (A003-1-2, A001-3-2, A006-2-1, Nanjing Jiancheng Technology Co., Ltd, China). RIPA lysis buffer (Thermo Scientific™, USA), The antibodies mentioned in the experiments included: Collagen II (abcam, ab34712), MMP-3 (abcam, ab52915), Nuclear factor erythroid 2-related factor 2 (NRF2, CST, 20733), Glutathione peroxidase 4 (GPX4, abcam, ab125066), Ferritin light chain 1 (FTL1, abcam, ab69090), Solute carrier family 7 member 11 (SLC7A11, abcam, ab175186), GAPDH (Proteintech, 60004-1-Ig), goat anti-rabbit IgG H&L (Beijing Zhong Shan-Golden Bridge Biological Technology Co.,Ltd, ZB-2301), goat anti-rat IgG H&L (Zhong Shan-Golden Bridge Biological Technology CO.,LTD, ZB-2305) ECL chemiluminescence reagent kit (Millipore, USA), NRF2 inhibitor ML385 (Selleck, S8790).

### Collection of human samples

This study was conducted in accordance with ethical guidelines and received approval from the Medical Ethics Committee of the Qingpu Branch of Zhongshan Hospital affiliated with Fudan University (approval code: qingyi2024-62). All patients provided informed consent. Nucleus pulposus specimens were collected from 9 patients who underwent surgical procedures between 2023 and 2024. The cohort included 5 females and 4 males, aged between 50 and 80 years. Prior to surgery, all patients underwent T2-weighted MRI scans to assess the degree of disc degeneration using the Pfirrmann grading system. Table 1 provides detailed information about the specimens utilized in this study.

**Table 1:** Details of clinical samples

Case	Gender	Age	Affected IVD	Pfirrmann grade
1	Male	53	L5/S1	III
2	Female	68	L3/4	IV
3	Female	79	L4/5	V
4	Male	70	L3/4	V
5	Male	56	L3/4	IV
6	Female	68	L4/5	IV
7	Male	79	L4/5	III
8	Male	65	L4/5	V
9	Male	62	L4/5	III

### Establishment of a rat IVDD model by acupuncture

Forty-eight male Sprague-Dawley rats (8 week) were applied for in vivo evaluation of ginsenoside Rg1. All animal protocols were approved by the Department of Animal Welfare and Ethics, Experimental Animal Science, Fudan University (approval code: 20240124 S). The rats were randomly divided into four groups: the control group, the IVDD group (receiving saline solution daily), the IVDD + ginsenoside Rg1-L group (receiving 20 mg/kg/day of Rg1), the IVDD + ginsenoside Rg1-H group (receiving 40 mg/kg/day of Rg1), with 12 rats in each group. The 36 SD rats were anesthetized by intraperitoneal injection of phenobarbital sodium (5 mg/100 g). After disinfection with iodinated polyvinyl pyrrolidone, a dorsal longitudinal skin incision was made. The Co7/8 disc was identified as the target segment. After fixation and disinfection, a longitudinal skin incision was made, and a syringe needle was inserted vertically (4–5 mm depth), rotated 360°, held for 30s, then removed before wound closure. Control rats received no puncture. This model effectively mimics human IVDD progression, inducing sustained severe degeneration within an appropriate timeframe while replicating the human degenerative cascade [23].

### Histopathology and Safranin O solid green staining

Intervertebral disc tissues were collected and stored in 10% neutral buffered formalin before being embedded in paraffin. Using a microtome, the paraffin-embedded specimens were sectioned into 5 μm slices. These sections were processed according to standard protocols and stained with hematoxylin and eosin (H&E) for histopathological analysis and safranin O solid green for the assessment of intervertebral disc morphology.

### Quantitative real-time polymerase chain reaction (qRT-PCR)

Total RNA was extracted using the Solarbio RNA extraction kit, followed by cDNA synthesis with the TransGen Biotech reverse transcription kit. Gene mRNA levels were quantified via qRT-PCR (TOLOBIO SYBR Master Mix) using primers listed in Table S1 (BLAST-verified). Data were normalized to GAPDH and analyzed by the 2^(−ΔΔCT)^ method.

### Western blot analysis

Total proteins were extracted from intervertebral disc NP tissues using RIPA buffer, separated by SDS-PAGE, and transferred to PVDF membranes. After blocking with 5% skimmed milk, membranes were incubated overnight at 4 °C with primary antibody, followed by 1 h secondary antibody incubation at room temperature. Protein bands were detected using ECL and analyzed with Image J.

### Immunohistochemical analysis

Immunofluorescence analysis was performed to investigate the localization and expression of target proteins in both cells (*n* = 3 per group) and IVDD tissue samples (*n* = 3 per group). NP tissue and cell sections were immunostained with Collagen II, MMP3, and NRF2 antibodies, incubated overnight at 4 °C, then treated with fluorescent secondary antibody (1 h, dark). After DAPI counterstaining, images were captured using a Nikon ECLIPSE Ni microscope. Images were captured and analyzed using image analysis software to quantify the fluorescence intensity. The relative expression levels and subcellular localization of the target proteins were compared between IVDD and control groups. Statistical analysis was performed to determine significant differences in protein expression and localization, with appropriate statistical tests employed to ensure rigor and reproducibility.

### Enzyme-linked immunosorbent assay (ELISA)

Serum was collected from rat abdominal aorta blood, centrifuged (4 °C, 10 min), and analyzed for MDA, SOD, and GSH-Px levels using ELISA kits according to the manufacturer’s protocol.

### X-Ray film and magnetic resonance imaging (MRI) analysis

Under anesthesia, rat tail X-rays were acquired (RADspeed M, Japan; 250 mA, 50 kV, 20ms) in the sagittal plane. Intervertebral disc height was measured using ImageJ and normalized to adjacent vertebrae to calculate the disc height index (DHI). MRI (GE 1.5T) with T2-weighted imaging (TR 3500ms, TE 120ms) assessed NP hydration. NP contours were delineated based on high signal intensity and analyzed using ImageJ. Four blinded researchers classified the disc images into five grades using the Pfirrmann classification system.

### Network pharmacological analysis

First, the target of Rg1 was found in PubChem database (https://pubchem.ncbi.nlm.nih.gov/) and the 2D structure was downloaded. The corresponding targets were retrieved from Swiss target prediction database (http://www.swisstargetprediction.ch/). GeneCards (https://www.genecards.org/) was selected as databases for disease target retrieval. Ferroptosis-related targets were screened from the FerrDB database (https://www.zhounan.org/ferrdb/). The retrieval results were combined and deduplicated. The overlapping genes of herbs and diseases were then used for the construction of the protein-protein interaction (PPI) network. Finally, import the PPI network into Cytoscape 3.7.2 software for visualization, and use CytoNCA plugin to analyze the preliminary network topology parameters to screen out core targets.

### Transcriptomic analysis

Transcriptomic analysis was conducted by Wuhan Saiweier Biotechnology Co., Ltd. NP tissues were randomly selected from rats in the Control, IVDD and Rg1-H groups (*n* = 3). The process involved enriching mRNA with polyA structure in total RNA using Oligo (DT) magnetic beads. Subsequently, the first strand of cDNA was synthesized in the M-MuLV reverse transcriptase system. The double-stranded cDNA was purified, subjected to end repair, add A-tail addition, and sequencing adapter ligation. Following further purification, PCR amplification was performed. Differential gene expression analysis was conducted using DESeq, with differentially expressed genes identified based on the criteria of expression fold change (|log2FoldChange| >1) and statistical significance (*p* value < 0.05).

### Molecular docking

The 2D structures of active compounds were obtained from PubChem (https://pubchem.ncbi.nlm.nih.gov/), while core targets (Homo sapiens) were retrieved from the PDB database (https://www.rcsb.org/), selecting high-resolution crystal structures with original ligands. Molecular docking was performed using Autodock, with results visualized and analyzed in PyMOL to generate interaction models.

### Isolation of rat primary NP cells

NP tissues were isolated from caudal IVDs (Co1-Co6) of euthanized 8-week-old SD rats (pentobarbital sodium, 50 mg/kg). After collagenase II digestion (2 mg/ml, 37 °C, 2 h), cells were centrifuged, resuspended, and cultured in DMEM with 15% FBS.

### Cell proliferation assay

Log-phase NP cells were trypsinized, counted (5 × 10⁴ cells/ml), and seeded in 96-well plates (5 × 10³ cells/well, triplicates). The plates were cultured at 37℃ overnight. Hydrogen peroxide (H_2_O_2_) can induce an IVDD model in human NP cells. H_2_O_2_ rapidly promoted ROS production and DNA damage in NP cells of human [24]. The screening drug concentrations for H_2_O_2_ were 10µM, 50µM, 100µM, 200µM, and 400µM. The screening drug concentrations for ginsenoside Rg1 were 10µM, 50µM, 100µM, 200µM, 400µM and 800µM. The plates were cultured for 48 h, after which a fresh culture medium containing a 10% Cell Counting Kit-8 (CCK-8) solution was added to the cells and incubated at 37℃ for 1 h. The optical density 450 nm was detected using a multimode microplate reader (Beijing Stawo Technology Co., Ltd, China).

### Lipid ROS and ferroorang staining

To assess ferroptosis, log-phase NP cells (5 × 10^5^ cells/ml) were seeded in 6-well plates. After 24 h adhesion, cells were stained with 10 µM DCFH-DA or 5 µM C11 BODIPY (20 min, dark), then analyzed by flow cytometry (480/525 nm excitation/emission). ROS-positive cells showed FITC-channel fluorescence.

### Immunofluorescence analysis

NP cells were immunostained for Collagen II, MMP3, and GPX4. After PBS washing and 4% formaldehyde fixation (30 min), cells were permeabilized (0.5% Triton X-100, 10 min), incubated with primary antibodies (4 °C, overnight) and fluorescent secondary antibodies (1 h, dark), then counterstained with DAPI. Images were captured using a Nikon ECLIPSE Ni microscope.

### Transmission electron microscope (TEM)

To observe the morphology of mitochondria, the cells were rapidly fixed with an electron microscope fixative solution at 4 °C for 2 to 4 h. After washing with 0.1 M PBS, the cells were fixed with a 2.5% glutaraldehyde fixed solution at room temperature for 2 h. Following a gradient dehydration process, the cells were embedded, sectioned, and then stained with 3% uranyl acetate and lead citrate for 15 min. Transmission electron microscopy (Hitachi SU8100) was utilized for image acquisition and analysis.

### Chromatin Immunoprecipitation (ChIP) assay

ChIP assay was undertaken using EZ ChIP Chromatin Immunoprecipitation Kit (Millipore, Billerica, MD, USA) following the manufacturer’s protocols. Briefly, 2 × 10^7^ cells were used for each individual reaction. The rat NP cells were fixed by 1% formaldehyde and lysed. The genome was sonicated 200 ~ 1000 bp DNA fragments. Ten microgram antibodies specific for NRF2 (CST, #20733, USA) were added into the cell lysate for incubation at 4 ℃ overnight, than incubated with Protein A Agarose/Salmon Sperm DNA beads (50% Slurry). The input DNA and immunoprecipitated DNA was purified and detected by qPCR using the primer sequences (Supplementary materials 1).

### Statistical analysis

All data were processed with Paragraph Prism 9.0.0 (GraphPad Software, La Jolla, CA) software. All data were represented as mean ± standard deviation (SD) and *p* < 0.05 was considered statistically different. Statistical analysis was performed by one-way analysis of variance (ANOVA) tests. Each experiment was repeated at least three times independently to confirm the consistency of the findings. The level of statistical significance was set at *p* < 0.05 for all analyses. Significance levels in the figures were denoted as follows: *p* < 0.05 (*), *p* < 0.01 (**), *p* < 0.001 (***), and *p* < 0.0001 (****). Non-significant results were denoted as “ns”.

## Results

### Ferroptosis in degenerated human NP tissue

**Fig. 1:**
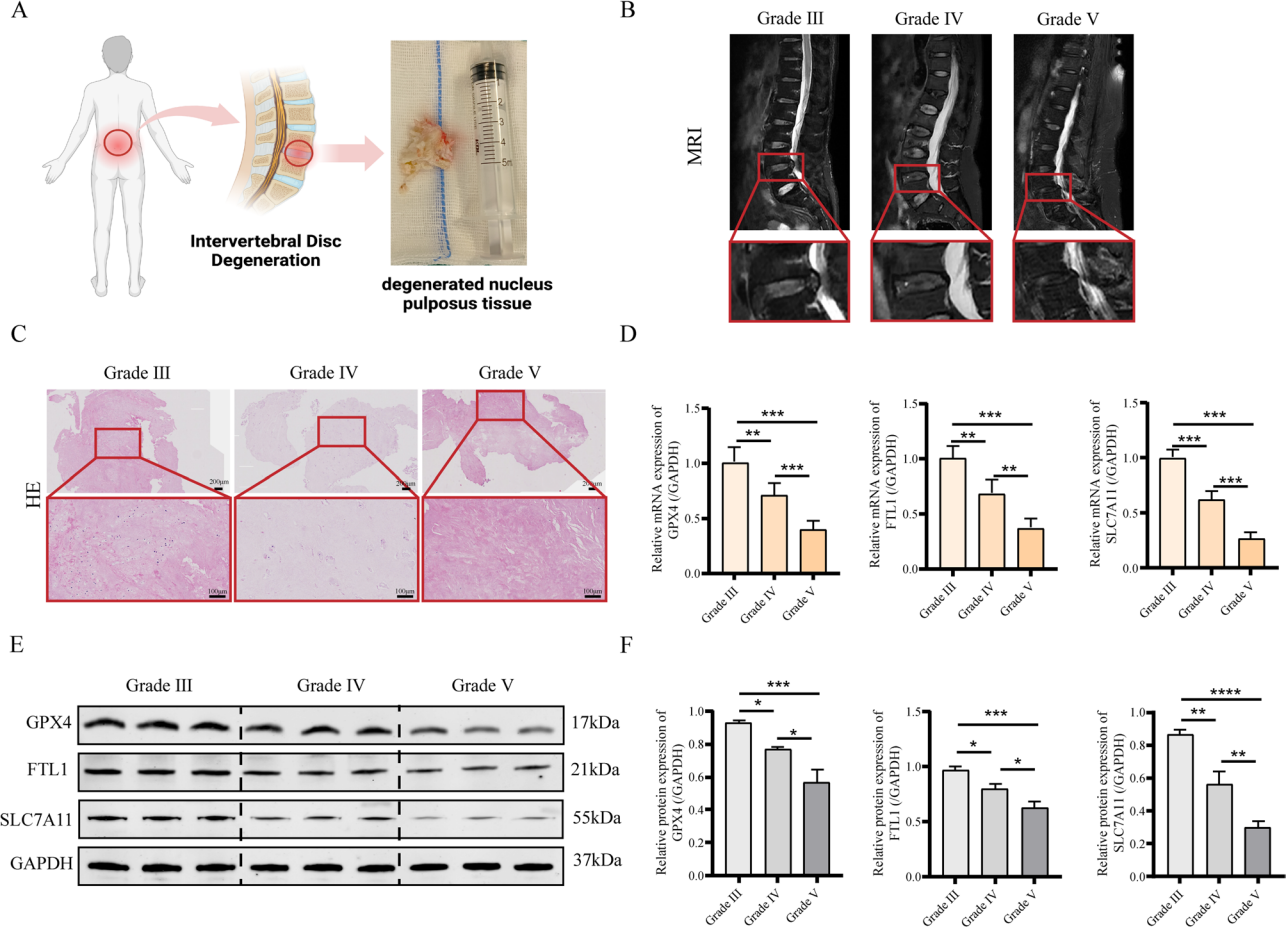
Ferroptosis in degenerated human NP tissue. (A) Schematic illustration of sampling degenerated NP tissue and human degenerated NP tissue, illustration created using BioRender.com. (**B**) Magnetic resonance imaging of the human lumbar spine and Pfirrmann grading. (**C**) Histopathology of human degenerated NP tissue (*n* = 3, scale bar:100 μm and 200 μm). (**D–F)** mRNA and protein expression levels of FTL1, GPX4, and SLC7A11 in human degenerated NP tissue (*n* = 3; one-way ANOVA). Data are presented as mean ± SD. Statistical significance is denoted as follows: *p < 0.05, **p < 0.01, ***p < 0.001, ****p < 0.0001

To investigate ferroptosis in IVDD, NP tissues from 9 patients across Pfirrmann grades were analyzed. MRI (Fig. 1A-B) and histopathology (Fig. 1C) revealed degenerative changes. qRT-PCR and WB showed progressive downregulation of GPX4, FTL1, and SLC7A11 with increasing degeneration (Fig. 1D-F), demonstrating ferroptosis’ critical role in human IVDD progression.

### Ginsenoside Rg1 could effectively mitigate in rats with IVDD and promote intervertebral disc repair

**Fig. 2:**
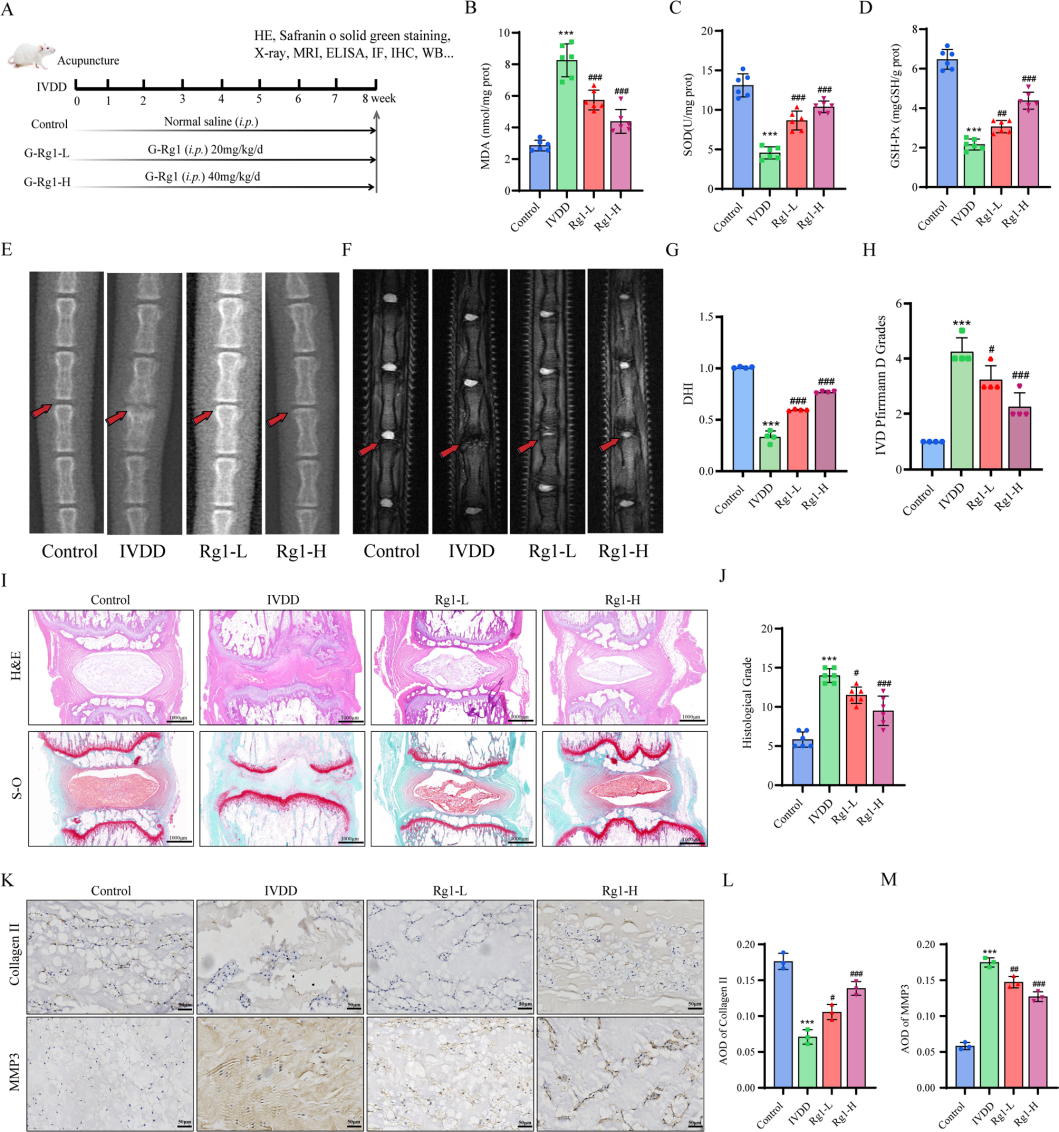
Ginsenoside Rg1 could effectively ameliorate in rats with IVDD and promote intervertebral disc repair. (**A**) The diagram of animal experiments. (**B–D**) Content of MDA, SOD and GSH (*n* = 3; one-way ANOVA). (**E–H)** X-ray and MRI examination (*n* = 3; one-way ANOVA). (**I, J**) Histopathology and safranin O solid green staining pathological analysis (*n* = 6, scale bar:1000 μm) (*n* = 3; one-way ANOVA). (K-M) Immunohistochemical staining of Collagen II and MMP3 in rat intervertebral disc (*n* = 3; one-way ANOVA). Data are presented as mean ± SD. Statistical significance is denoted as follows: ***p < 0.001 VS Control; #*p* < 0.05, ##*p* < 0.01, ###*p* < 0.001 VS IVDD

A detailed flowchart of the in vivo assays was shown in Fig. 2A. Ginsenoside Rg1 treatment significantly reduced MDA levels (Fig. 2B) and restored SOD/GSH activity (Fig. 2C, D) in IVDD rats. Calculation of the disc height index (DHI) showed that the degenerated intervertebral disc height of IVDD group decreased over 70%. Compared with the control group, and the ginsenoside Rg1 group had less disc height loss, restored the height to about 50% of the control group (Fig. 2E, G). The MRI results showed that the nucleus pulposus and endplates exhibited a black color in the IVDD group, while the Pfirrmann grade was elevated. In contrast to these findings, the Pfirrmann grade decreased in the ginsenoside Rg1-H and ginsenoside Rg1-L groups, the endplate gradually became white, and the signal gradually recovered (Fig. 2F, H). The results of HE staining and safranin O solid green staining revealed a significant reduction in the presence of NP tissue within the IVDD group, accompanied by a pronounced severity of fibrosis. However, the intervertebral disc nucleus pulposus in the ginsenoside Rg1 intervention group was relatively full (Fig. 2I, J). Immunohistochemistry demonstrated Rg1 upregulated Collagen II while downregulating MMP3 (Fig. 2K-M), confirming its protective effect against IVDD progression. Thus, ginsenoside Rg1 can inhibit IVDD progression in the rat tail puncture model.

### The network Pharmacology and transcriptomics of ginsenoside Rg1 on alleviating IVDD

**Fig. 3:**
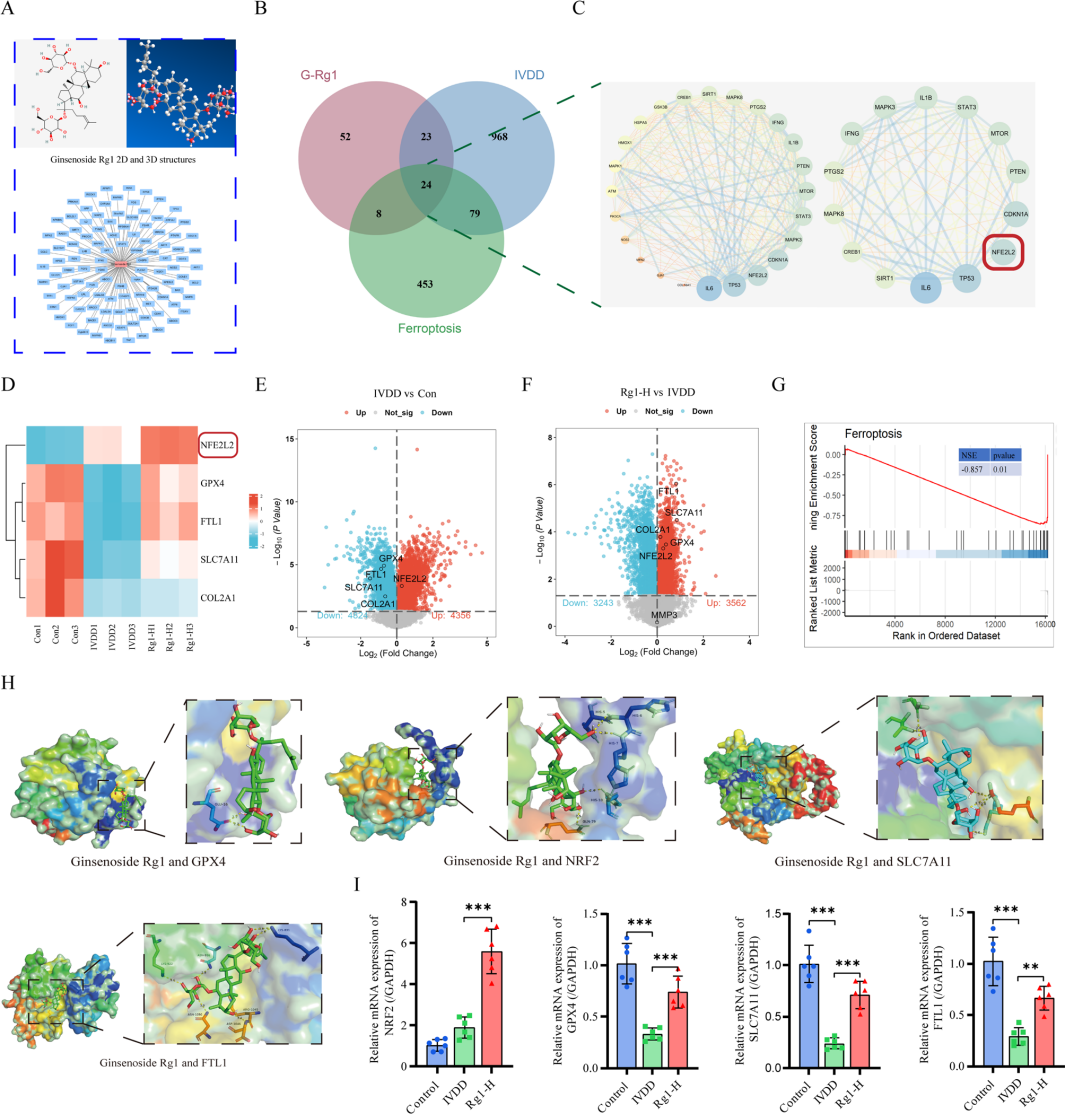
The network pharmacology and transcriptomics of ginsenoside Rg1 on alleviating IVDD. (**A**) Ginsenoside Rg1 2D structure and Ginsenoside Rg1-target map. (**B**) Ginsenoside Rg1 IVDD and Ferroptosis venn diagram. (**C**) PPI network and core targets. (**D**) Transcriptome Control vs. IVDD vs. Rg1-H and heatmaps. (**E**) Transcriptome Control vs. IVDD volcano plots. (**F**) Transcriptome Rg1-H vs. IVDD volcano plots. (**G**) Gene set enrichment analysis of ferroptosis in the sequencing of human degenerative IVD. (**H**) Molecular docking. (**I**) mRNA and protein expression levels of FTL1, GPX4, and SLC7A11 (*n* = 3; one-way ANOVA). Data are presented as mean ± SD. Statistical significance is denoted as follows: **p < 0.01,***p < 0.001

Through network pharmacology analysis, 107 potential Rg1 targets were identified (Fig. 3A), with 24 overlapping targets between IVDD, ferroptosis, and Rg1 (Fig. 3B). PPI network analysis (median Degree = 19, BC = 4.11, CC = 0.85) revealed 14 core targets including NRF2 (Fig. 3C). Transcriptome sequencing confirmed differential expression of ferroptosis-related genes (NRF2, GPX4, SLC7A11, FTL1) (Fig. 3D-F). GSEA showed ferroptosis genes were predominantly expressed in the IVDD group (Fig. 3G).

Integrated network pharmacology and transcriptomics identified NRF2 as the key target through which ginsenoside Rg1 may ameliorate IVDD by regulating ferroptosis. Molecular docking revealed strong binding affinities between Rg1 and ferroptosis-related proteins: NRF2 (− 7.4 kcal/mol), GPX4 (− 5.6 kcal/mol), SLC7A11 (− 9.1 kcal/mol), and FTL1 (− 8.5 kcal/mol) (Fig. 3H) (Table S2). PCR analysis demonstrated that Rg1 significantly upregulated NRF2 expression in IVDD rats (Fig. 3I) while reversing the IVDD-induced downregulation of GPX4, SLC7A11, and FTL1, suggesting its therapeutic potential through modulation of these critical ferroptosis pathway components.

### Ginsenoside Rg1 ameliorated rats with IVDD via inhibiting ferroptosis

**Fig. 4:**
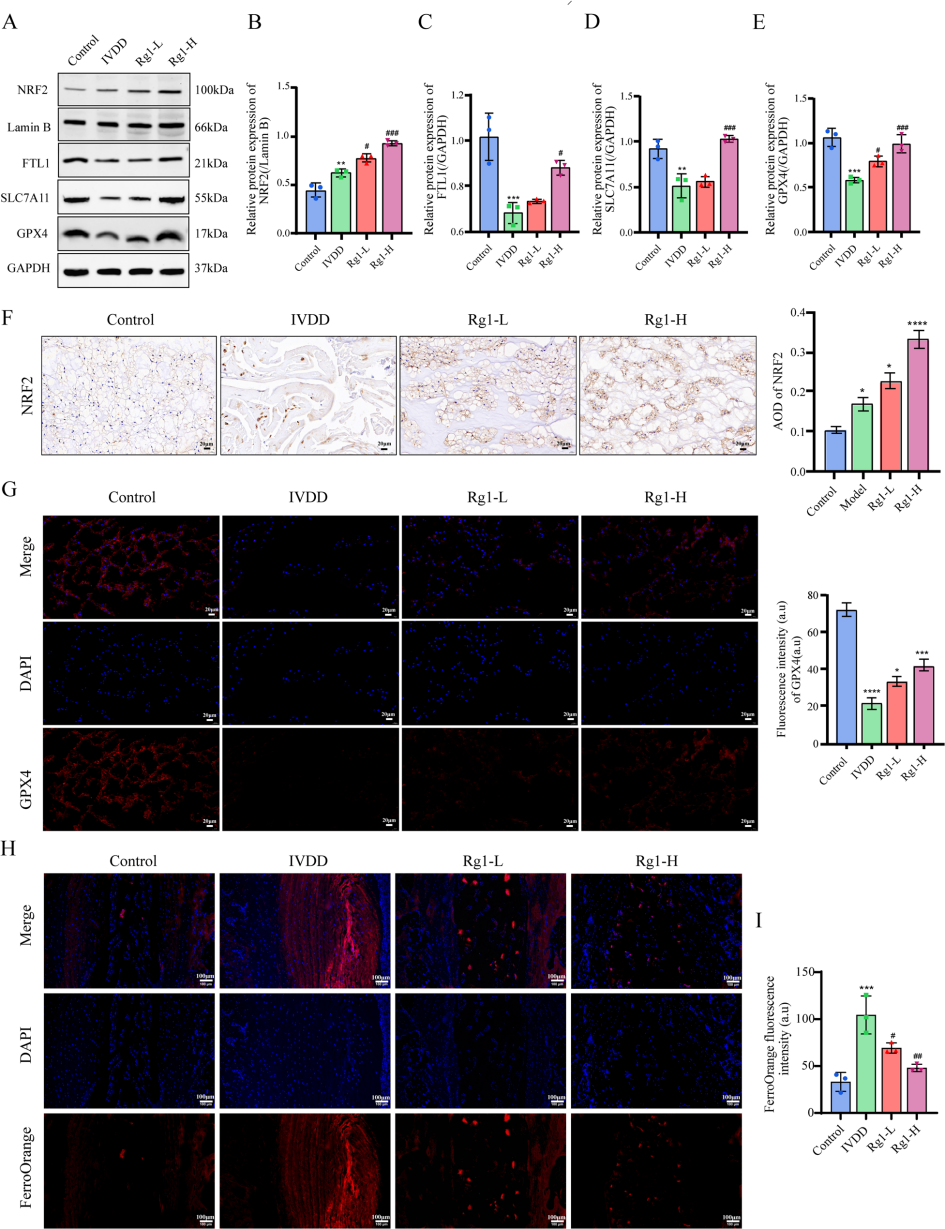
Ginsenoside Rg1 ameliorated IVDD of the rats via inhibiting ferroptosis. (**A–E**) Western Blot detection of NRF2, GPX4, FTL1, and SLC7A11 protein levels (*n* = 3; one-way ANOVA). (**F**) Immunohistochemistry detection of NRF2 expression (*n* = 3, scale bar:20 μm) (*n* = 3; one-way ANOVA). (**G**) Immunofluorescence detection of GPX4 expression (*n* = 3, scale bar:20 μm) (*n* = 3; one-way ANOVA). (**H, I**) Observation of free Fe^2+^ levels in rats with IVDD using FerroOrange probe (*n* = 3, scale bar:100 μm). Data are presented as mean ± SD. Statistical significance is denoted as follows: **p < 0.01, ***p < 0.001 VS Control; #*p* < 0.05, ###*p* < 0.001 VS IVDD

NRF2 is essential for the defense against ferroptosis. WB and immunohistochemistry confirmed ginsenoside Rg1 upregulated NRF2 expression in IVDD rats (Fig. 4A, B,F). As NRF2’s downstream ferroptosis targets, GPX4, FTL1 and SLC7A11 protein levels were significantly restored by Rg1 treatment (Fig. 4A-E). Immunofluorescence demonstrated enhanced GPX4 expression (Fig. 4G), while FerroOrange assays showed Rg1 reduced Fe^2+^ accumulation (Fig. 4H, I), collectively indicating Rg1’s anti-ferroptotic effect through NRF2 pathway activation.

### Ginsenoside Rg1 ameliorated H_2_O_2_-induced NP cells degeneration

**Fig. 5:**
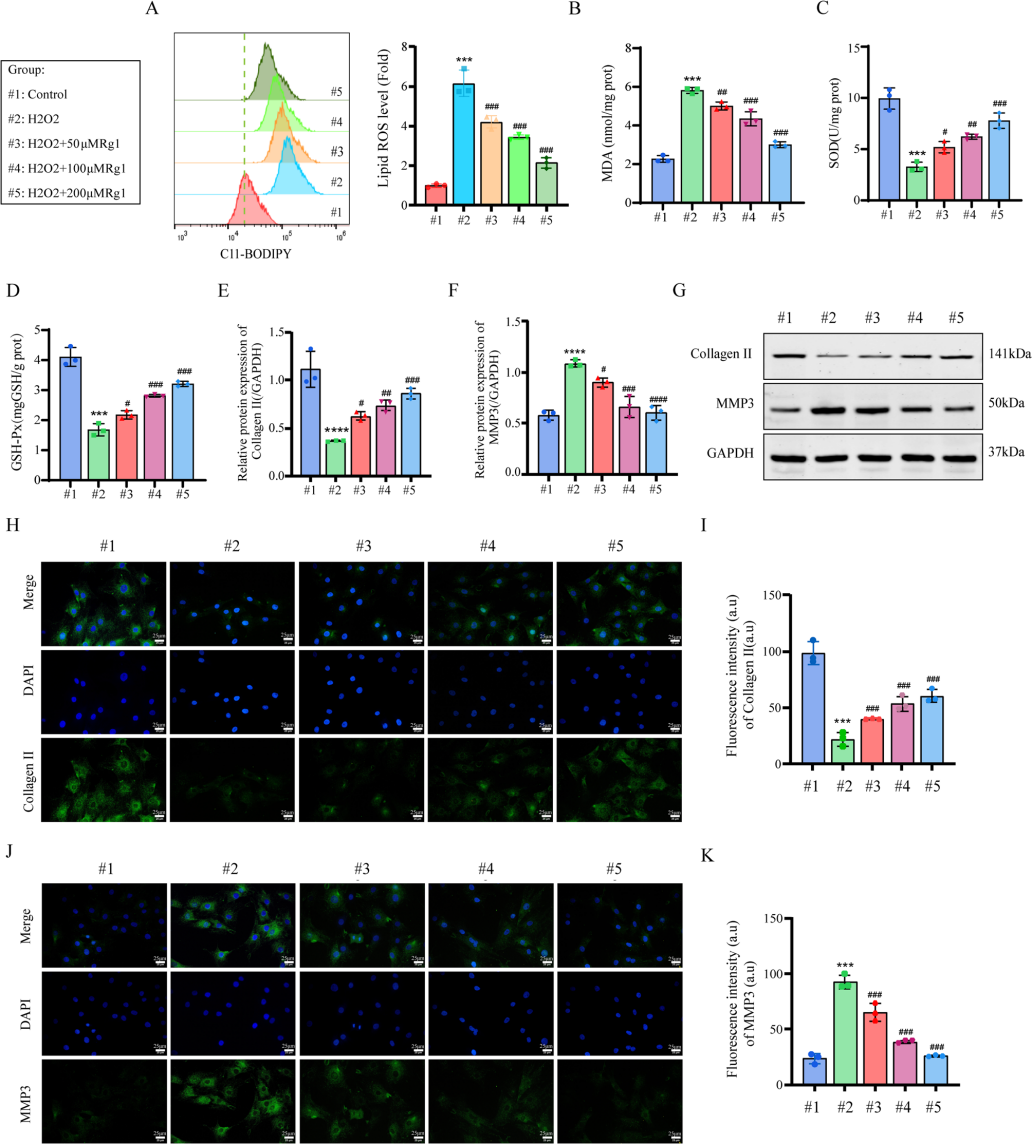
Ginsenoside Rg1 ameliorated H_2_O_2_-induced NP degeneration. (**A**) Flow cytometry detection of Lipid-ROS levels in NP cells (*n* = 3; one-way ANOVA). (**B–D)** Content of MDA, SOD and GSH (*n* = 3; one-way ANOVA). (**E–G)** Western Blot detection of Collagen II and MMP3 protein levels (*n* = 3; one-way ANOVA). (**H–K**) Immunofluorescence detection of protein levels of Collagen II and MMP3 (*n* = 3, scale bar:25 μm). Data are presented as mean ± SD. Statistical significance is denoted as follows: ***p < 0.001, ****p < 0.0001 VS Control; #*p* < 0.05, ##*p* < 0.01, ###*p* < 0.001, ####*p* < 0.0001 VS H_2_O_2_

Integrated analyses identified ferroptosis as crucial in IVDD (Fig. 3). Treatment with 200 µM Rg1 effectively normalized Lipid-ROS levels (Fig. 5A), reduced MDA (Fig. 5B), and restored SOD/GSH-Px activity (Fig. 5C, D). WB and immunofluorescence demonstrated Rg1 reversed H_2_O_2_-induced ECM dysregulation by increasing Collagen II and decreasing MMP3 expression (Fig. 5E-K), confirming its protective role in NP cell homeostasis.

### Ginsenoside Rg1 ameliorated H_2_O_2_-induced NP cells degeneration by suppressing ferroptosis

**Fig. 6:**
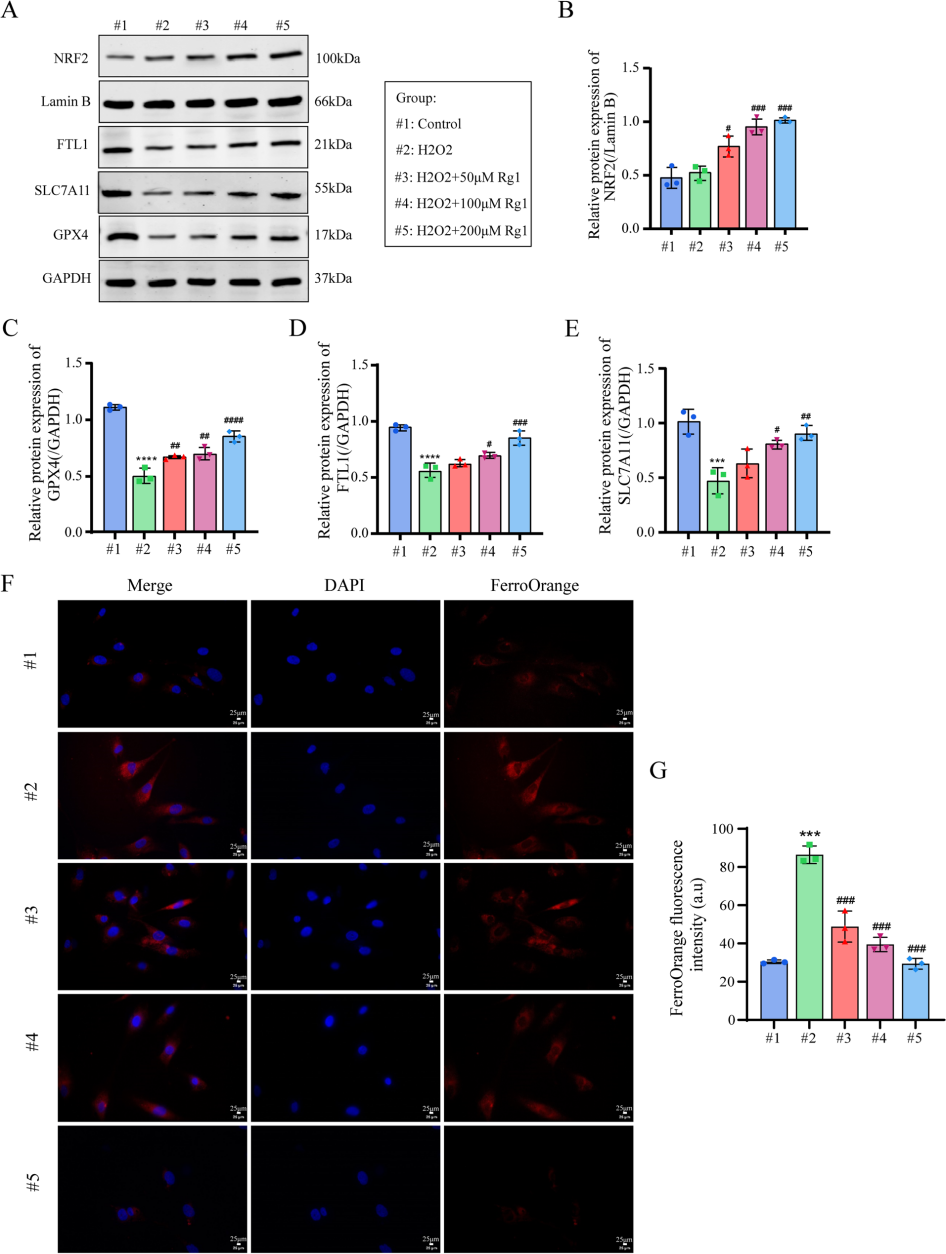
Ginsenoside Rg1 ameliorated H_2_O_2_-induced NP cells degeneration by suppressing ferroptosis. (**A–E**) Western Blot detection of NRF2, GPX4, FTL1, and SLC7A11 protein levels (*n* = 3; one-way ANOVA). (**F, G**) Observation of free Fe^2+^ content in NP cells using FerroOrange probe (*n* = 3, scale bar:25 μm). Data are presented as mean ± SD. Statistical significance is denoted as follows: ***p < 0.001, ****p < 0.0001 VS Control; #*p* < 0.05, ##*p* < 0.01, ###*p* < 0.001 VS H_2_O_2_

NRF2 and GPX4 are key oxidative stress sensors in NP cells and play pivotal roles in ferroptosis. Network pharmacology and transcriptome analysis identified FTL1 and SLC7A11 as additional ferroptosis-related targets. NRF2, a critical ferroptosis suppressor, mitigates ferroptosis by upregulating downstream targets. WB results revealed elevated NRF2 levels in IVDD NP cells, further increased by ginsenoside Rg1 treatment (Fig. 6A, B). GPX4, negatively regulating ferroptosis, prevents lipid peroxide accumulation; its decline in H_2_O_2_-induced NP cells was reversed by Rg1. Reduced FTL1 (causing Fe^2+^ overload) and decreased SLC7A11 (a ferroptosis inhibitor) in H_2_O_2_-treated cells were also restored by Rg1 (Fig. 6A-E). Fe^2+^ probe assays confirmed that 50/100/200µM Rg1 significantly reduced iron overload in H_2_O_2_-stimulated NP cells after 48 h (Fig. 6F, G).

### Ginsenoside Rg1 ameliorated NP cells activity by activating NRF2/GPX4 signaling pathway

**Fig. 7:**
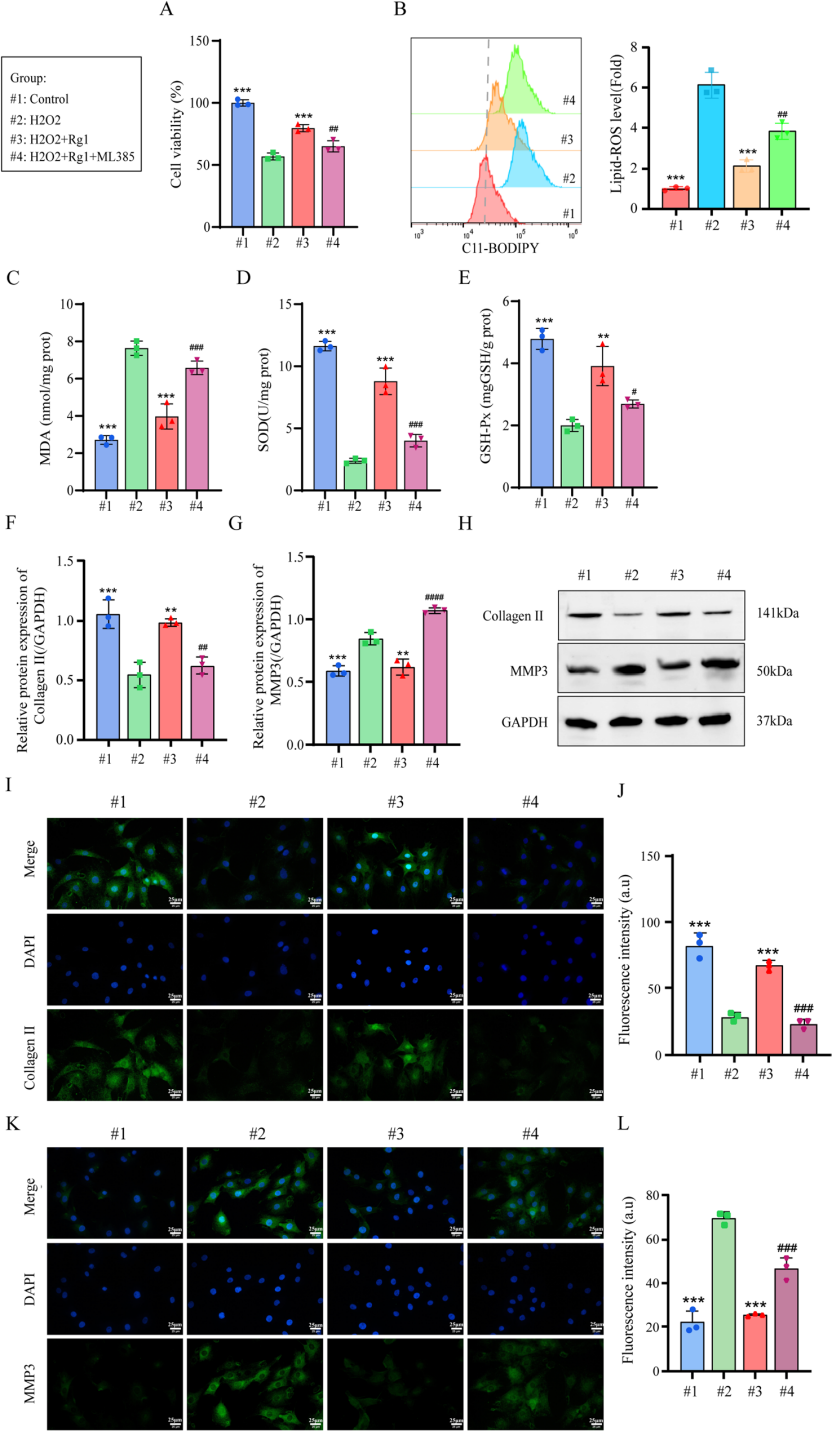
Ginsenoside Rg1 ameliorated NP cells activity by activating NRF2/GPX4 Signaling Pathway. NRF2 inhibitor ML385 pre-treatment of H_2_O_2_-induced NP cells. (**A**) CCK8 detection of cell activity (*n* = 3; one-way ANOVA). (**B**) Flow cytometry detection of Lipid-ROS levels in cells (*n* = 3; one-way ANOVA). (C-E) Detection of MDA, SOD, and GSH content in cells (*n* = 3; one-way ANOVA). (**F–H**) Western Blot detection of Collagen II and MMP3 protein levels (*n* = 3; one-way ANOVA). (**I, J**) Immunofluorescence detection of protein levels of Collagen II (*n* = 3, scale bar:25 μm). (**K, L**) Immunofluorescence detection of protein levels of MMP3 (*n* = 3; one-way ANOVA). Data are presented as mean ± SD. Statistical significance is denoted as follows: ***p < 0.001 VS H_2_O_2_; #*p* < 0.05, ##*p* < 0.01, ###*p* < 0.001 VS H_2_O_2_+Rg1

The NRF2 inhibitor ML385 reduced NP cell viability (Fig. 7A) and increased Lipid-ROS accumulation (Fig. 7B). Co-treatment with ginsenoside Rg1 + ML385 elevated MDA levels while suppressing SOD and GSH-Px activity (Fig. 7C-E). Although Rg1 alone upregulated Collagen II and downregulated MMP3 in H_2_O_2_-induced NP cells, ML385 reversed these effects (Fig. 7F-H). Immunofluorescence confirmed that ML385 counteracted Rg1’s benefits, reducing Collagen II (Fig. 7I, J) and increasing MMP3 (Fig. 7K, L). These results demonstrate that ginsenoside Rg1’s protective effects against H_2_O_2_-induced damage in NP cells are NRF2-dependent.

### Ginsenoside Rg1 inhibited ferroptosis to ameliorate NP cells activity via activating NRF2

**Fig. 8:**
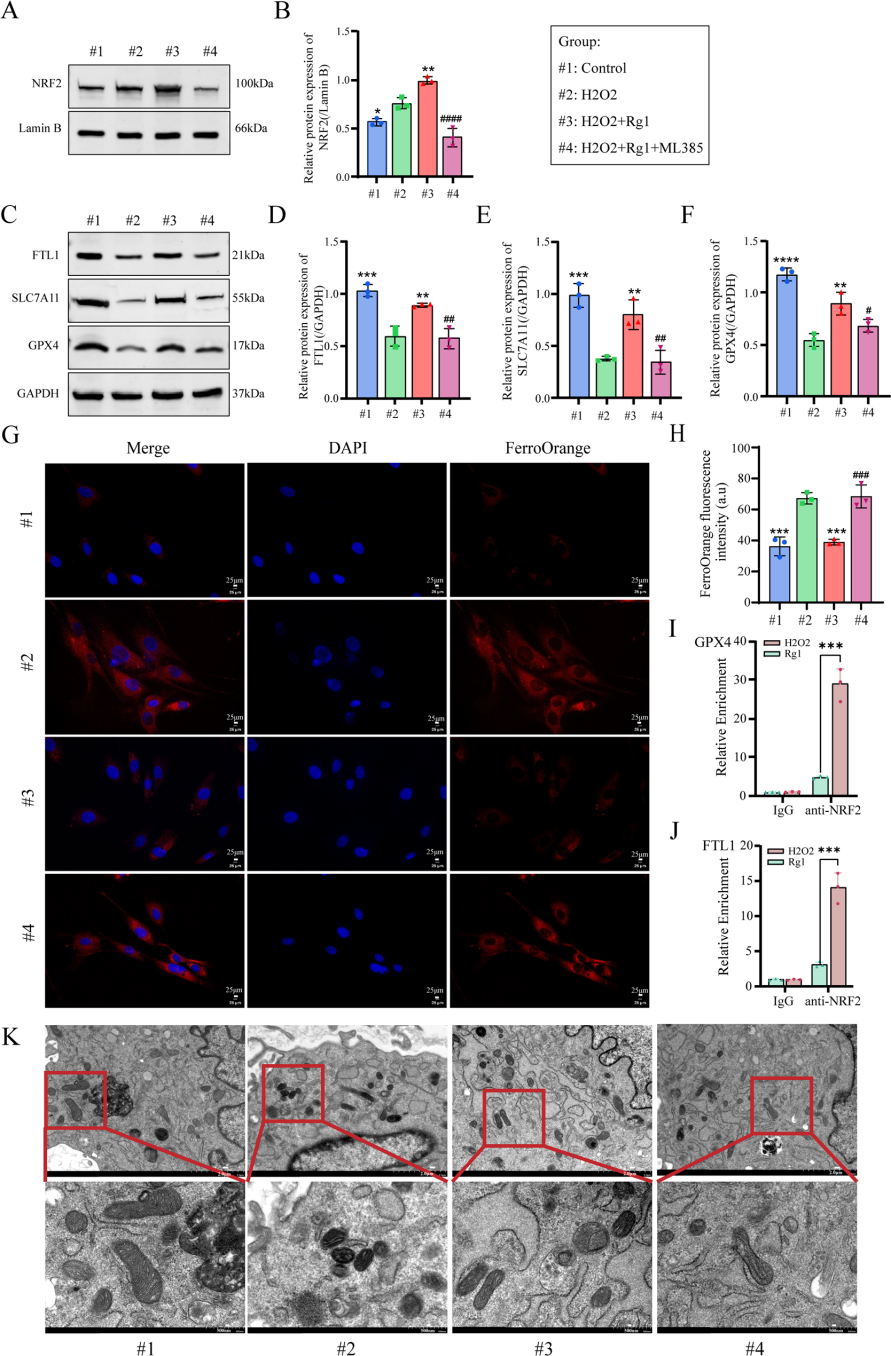
Ginsenoside Rg1 inhibited ferroptosis to ameliorate NP cells activity via activating NRF2. NRF2 inhibitor ML385 pre-treatment of H_2_O_2_-induced NP cells. (**A, B**) Western Blot detection of protein levels of NRF2 in the nucleus (*n* = 3; one-way ANOVA). (**C–F**) Western Blot detection of protein levels of ferroptosis key genes GPX4, FTL1, and SLC7A11 (*n* = 3; one-way ANOVA). (**G–H)** Observation of free Fe^2+^ levels in NP cells using FerroOrange probe (*n* = 3, scale bar:25 μm). (**I**) Observation of the morphology of mitochondria in nucleus pulposus using a transmission electron microscope (*n* = 3, scale bar:500 nm). Data are presented as mean ± SD. Statistical significance is denoted as follows: *p < 0.05, ***p < 0.001, ****p < 0.0001 VS H_2_O_2_; #*p* < 0.05, ##*p* < 0.01, ###*p* < 0.001, ####*p* < 0.0001 VS H_2_O_2_+Rg1

NRF2 inhibition by ML385 significantly decreased NRF2 protein levels (Fig. 8A, B) and downregulated ferroptosis-related proteins GPX4, FTL1, and SLC7A11 (Fig. 8C-F). Fe²⁺ probe analysis revealed elevated iron levels in the Rg1 + ML385 group compared to Rg1 alone, indicating ferroptosis reactivation (Fig. 8G, H). ChIP assays demonstrated that Rg1 enhanced NRF2’s binding to GPX4 and FTL1 promoters, promoting their transcription (Fig. 8I-J). TEM analysis showed that Rg1 rescued H_2_O_2_-induced mitochondrial damage (shrinkage, cristae condensation, and membrane rupture), but ML385 abolished this protective effect (Fig. 8K), confirming NRF2’s essential role in Rg1-mediated ferroptosis suppression and mitochondrial preservation.

## Discussion

Recent advances in medical research have led to gradual improvements in the treatment of intervertebral disc degeneration. Drug therapy and surgical treatments are effective in clinical practice, but they are often accompanied by high recurrence rates, major bleeding, and other complications. Therefore, alleviating IVDD remains a significant research focus.

This study observed significant ferroptosis in the NP tissue of patients with IVDD, with more pronounced ferroptosis in patients with higher imaging grades, indicating that ferroptosis plays a crucial role in IVDD progression. Panax ginseng C.A.Mey., a traditional Chinese medicine, has been widely used, and one of its main components, Ginsenoside Rg1, shows great potential in treating IVDD [11, 18]. To elucidate the mechanism of action of ginsenoside Rg1 in alleviating IVDD, network pharmacology and transcriptome sequencing were utilized to predict potential molecular targets [25]. Gene enrichment analysis revealed that ferroptosis is highly enriched in degenerative intervertebral disc tissue, including genes such as NRF2, indicating its critical role in IVDD. Ferroptosis can result from oxidative stress [26]. When the intracellular GSH antioxidant system fails to detoxify these species in a timely manner, it leads to lipid peroxidation and ferroptosis [27]. NRF2, a well-known transcription factor, plays a crucial role in antioxidant activity [28]. NRF2 can affect ferroptosis by regulating the gene expression of the GSH antioxidant system and iron (Fe) metabolism. Downstream targets of NRF2 include GPX4, GSH-transferase, and GSH reductase, among others [28]. Therefore, ferroptosis was tested in both in vitro and in vivo experiments.

Building upon previous research, we constructed a rat IVDD model using acupuncture to evaluate the therapeutic effect of ginsenoside Rg1 [29]. We induced IVDD using a 21-gauge needle puncture (5 mm depth, 30 s) at Co7/8, creating controlled annular injury that mimics early human IVDD through mechanical disruption, ECM catabolism, and oxidative stress [23, 30]. Untreated rats developed Pfirrmann grade III-V degeneration, matching human chronic IVDD specimens. Rg1 treatment effectively preserved NP matrix integrity during the acute phase and mitigated chronic disc degeneration, as evidenced by improved disc height index (DHI) on X-ray and enhanced hydration on T2-weighted MRI. Our study uniquely demonstrates Rg1’s therapeutic potential across both acute and chronic IVDD phases. While these findings highlight Rg1’s reparative capacity, future studies should incorporate more detailed temporal analyses to fully characterize its stage-dependent effects. We observed a significant reduction in the NP tissue within the IVDD group, associated with substantial fibrosis. The decrease in the nuclear matrix protein Collagen II and the increase in MMP3, a key gene for ECM synthesis and catabolism, suggest that IVDD in rats is challenging to repair. Elevated levels of lipid peroxide degradation products, such as MDA, and decreased activity of oxidative stress-related factors, including SOD and GSH, exacerbated the condition in rats. Additionally, X-ray and MRI results demonstrated high degeneration in IVDD rat discs, with the NP and endplates appearing black. Following ginsenoside Rg1 intervention, IVDD was alleviated and repaired. Using H_2_O_2_-induced NP cell degeneration, we evaluated the therapeutic efficacy of ginsenoside Rg1 in vitro [31]. In these cell experiments, ginsenoside Rg1 reduced ROS levels in rat NP cells, decreased MDA content, and increased SOD and GSH-Px activity, consistent with in vivo results.

Based on these findings, we further investigated the therapeutic mechanism of ginsenoside Rg1 in alleviating IVDD. Our studies confirmed that ginsenoside Rg1 increased the gene expression of NRF2 and upregulated GPX4, FTL1, and SLC7A11. Additionally, Fe^2+^ content accumulated in the nucleus tissue and cells of rats with IVDD, and ginsenoside Rg1 reduced this Fe^2+^ content as detected by FerroOrange probe fluorescence. When the NRF2 inhibitor ML385 was added to rat nucleus pulposus cells, the therapeutic effects of ginsenoside Rg1 were reversed. The NRF2 inhibitor group not only downregulated ferroptosis-related proteins (NRF2, GPX4, FTL1 and SLC7A11) but also promoted Fe^2+^ accumulation and lipid peroxidation in NP cells, increased MDA production, decreased SOD and GSH-Px activity under oxidative stress, damaged mitochondrial morphology and function, and aggravated IVDD. This demonstrated that ginsenoside Rg1 protected against IVDD by inhibiting ferroptosis in NP cells through the NRF2/GPX4 pathway.

Ginsenoside Rg1, a natural product extracted from Panax ginseng C.A.Mey., has demonstrated remarkable anti-inflammatory and antioxidant effects. Recent studies have elucidated that ginsenoside Rg1 mitigates acute ulcerative colitis by modulating gut microbiota and microbial tryptophan metabolism [32]. Furthermore, it ameliorates sepsis-induced acute kidney injury by inhibiting ferroptosis in renal tubular epithelial cells [22] and attenuates oxidative stress and inflammation in rats with spinal cord injury via the NRF2/HO-1 signaling pathway [33]. Additionally, ginsenoside Rg1 has been shown to inhibit IVDD progression by suppressing the activation of the YAP1/TAZ signaling pathway [11] and promoting ECM synthesis while inhibiting apoptosis in degenerative NP cells through the Wnt/β-catenin pathway [18]. This study provides novel insights into the preventive and therapeutic effects of ginsenoside Rg1 in alleviating IVDD and its inhibitory effect on ferroptosis in NP cells.

Emerging evidence implicates ferroptosis as a critical pathological mechanism in various bone-related disorders including osteoporosis, bone defects, osteoarthritis, and peri-implant bone loss [34–36]. Our study demonstrates that this potential mechanism: Ginsenoside Rg1-mediated ferroptosis suppression mitigates IVDD. Based on the above literature studies, we speculate that ferroptosis regulation serve as a shared therapeutic strategy for bone-related disorders characterized by redox imbalance.We plan to conduct in-depth studies on these aspects in our future research. The NRF2/GPX4 axis emerges as a promising therapeutic target, which has been demonstrated in a variety of bone-related disorders. In bone marrow mesenchymal stem cells, NRF2/GPX4 activation enhances osteogenic capacity under oxidative stress [37], while in chondrocytes, SIRT1/NRF2/HO-1 signaling mitigates osteoarthritis progression by suppressing ferroptosis [36]. Our results align with prior evidence, indicating that Rg1-induced NRF2/GPX4 pathway activation exerts cytoprotective effects on nucleus pulposus cells, which implies a universal regulatory mechanism in bone-related disorders.

This study has several limitations. First, the small sample size of human NP tissues (*n* = 9) may limit clinical relevance, despite using randomized selection to minimize variability. future studies should include larger cohorts. Second, while the acupuncture-induced rat IVDD model effectively replicates key degenerative features [30, 38], its translational relevance requires validation through comparison with other models and clinical studies. Third, although ginsenoside Rg1’s effect on ferroptosis appears NRF2-dependent, contributions from other pathways cannot be excluded. while pharmacological inhibition strongly suggests Nrf2 dependence, future studies using Nrf2 knockdown/knockout models would provide additional mechanistic confirmation. We are currently optimizing siRNA transfection protocols in primary NPCs for follow-up studies. While preclinical results are promising, clinical trials are needed to confirm ginsenoside Rg1’s therapeutic efficacy in human IVDD.

## Conclusion

**Fig. 9:**
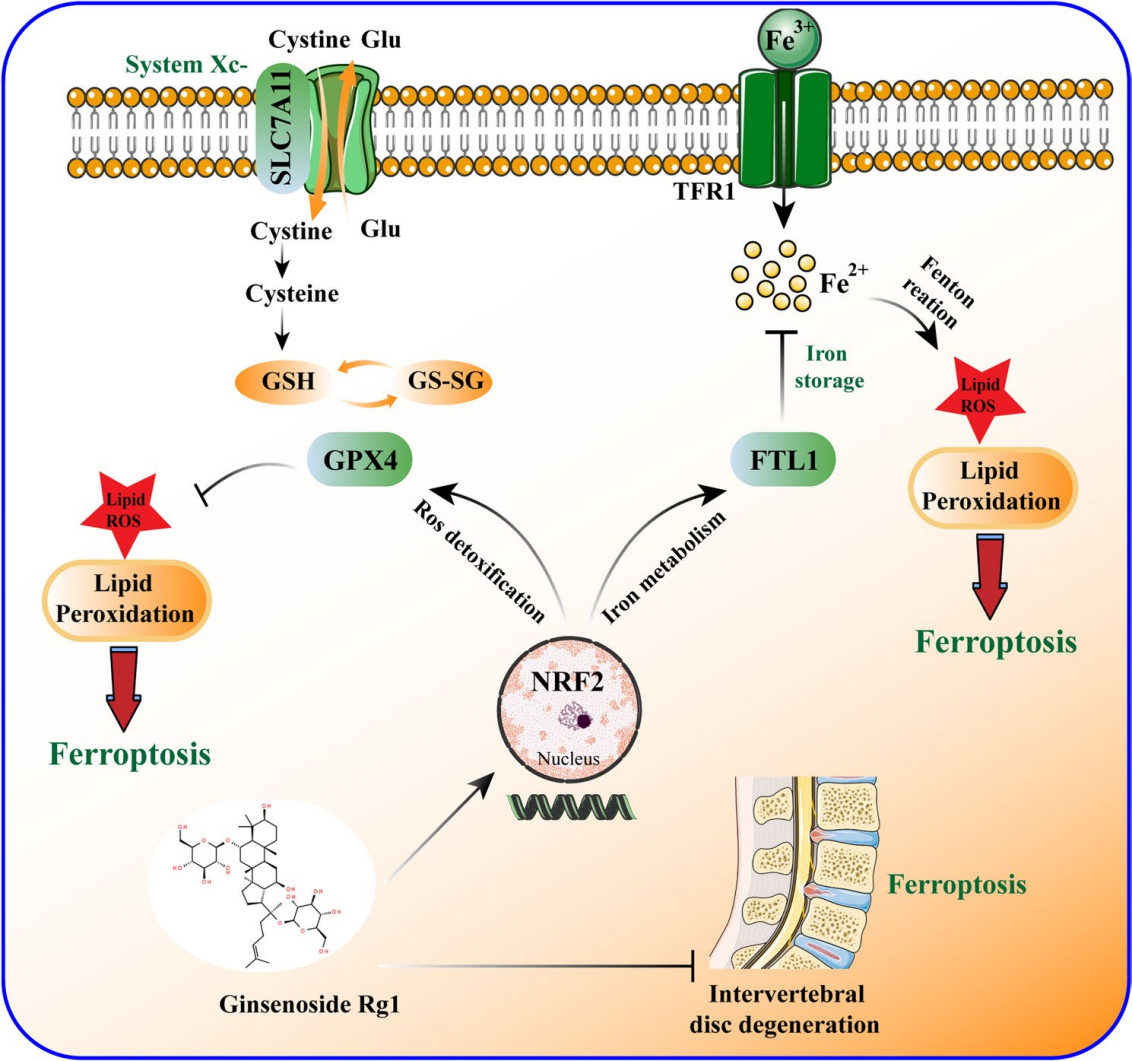
The molecular mechanism of ginsenoside Rg1 in alleviating IVDD

This study demonstrates that ginsenoside Rg1 exerts a protective effect against IVDD by suppressing ferroptosis in NP cells through the activation of NRF2 and regulation of GPX4. (Fig. 9). By elucidating the molecular mechanism underlying ginsenoside Rg1’s ability to inhibit ferroptosis in NP cells, this study provides valuable insights for the prevention and treatment of IVDD. These findings suggest that ginsenoside Rg1 may serve as a promising therapeutic agent for IVDD therapy by acting a ferroptosis inhibitor.

## Electronic supplementary material

Below is the link to the electronic supplementary material.

Supplementary Material 1

Supplementary Material 2

Supplementary Material 3

Supplementary Material 4

Supplementary Material 5

## Data Availability

The raw data supporting the conclusions of this article will be made available by the authors on request.

## Abbreviations

H_2_O_2_: Hydrogen peroxide
DHI: Disc height index
DCFH-DA: Dichlorodihydro-fluorescein diacetate
ECM: Extracellular matrix
ELISA: Enzyme-linked immunosorbent assay
FTL1: Ferritin light chain 1
GESA: Gene set enrichment analysis
GPX4: Glutathione peroxidase 4
GSH-Px: Glutathione Peroxidase
H&E: Hematoxylin and eosin
IVDD: Intervertebral disc degeneration
MDA: Malondialdehyde
MMPs: Matrix metalloproteinases
MRI: Magnetic resonance imaging
NP: Nucleus pulposus
NRF2: Nuclear factor erythroid 2-related factor 2
GPX4: Glutathione peroxidase 4
HO-1: Heme oxygenase 1
PPI: Protein-protein interaction
PVDF: Polyvinylidene fluoride
qRT-PCR: Quantitative real-time polymerase chain reaction
RCD: Regulated cell death
ROS: Reactive oxygen species
SDS-PAGE: Sodium dodecyl sulfate-polyacrylamide gel electrophoresis
SLC7A11: Solute carrier family 7 member 11
SOD: Superoxide Dismutase
ChIP: Chromatin immunoprecipitation assay
TEM: Transmission electron microscope

## References

[1] Xin J, Wang Y, Zheng Z, Wang S, Na S, Zhang S. Treatment of intervertebral disc degeneration. Orthop Surg. 2022;14:1271–80. 10.1111/os.13254. 35486489 10.1111/os.13254 PMC9251272

[2] Hu X, Wang Z, Zhang H, Cui P, Li Y, Chen X, Kong C, Wang W, Lu S. Single-cell sequencing: new insights for intervertebral disc degeneration. Biomed Pharmacother. 2023;165:115224. 10.1016/j.biopha.2023.115224. 37516017 10.1016/j.biopha.2023.115224

[3] Kang L, Zhang H, Jia C, Zhang R, Shen C. Epigenetic modifications of inflammation in intervertebral disc degeneration. Ageing Res Rev. 2023;87:101902. 10.1016/j.arr.2023.101902. 36871778 10.1016/j.arr.2023.101902

[4] Kritschil R, Scott M, Sowa G, Vo N. Role of autophagy in intervertebral disc degeneration. J Cell Physiol. 2022;237:1266–84. 10.1002/jcp.30631. 34787318 10.1002/jcp.30631 PMC8866220

[5] Capelletti MM, Manceau H, Puy H. K peoc’h. Ferroptosis in liver diseases: an overview. Int J Mol Sci. 2020;21:4908. 10.3390/ijms21144908. 32664576 10.3390/ijms21144908 PMC7404091

[6] Tu J, Li W, Yang S, Yang P, Yan Q, Wang S, Lai K, Bai X, Wu C, Ding W, Cooper-White J, Diwan A, Yang C, Yang H, Zou J. Single-Cell transcriptome profiling reveals multicellular ecosystem of nucleus pulposus during degeneration progression. Adv Sci (Weinh). 2022;9:e2103631. 10.1002/advs.202103631. 34825784 10.1002/advs.202103631 PMC8787427

[7] Zhou L, Cai F, Zhu H, Xu Y, Tang J, Wang W, Li Z, Wu J, Ding Z, Xi K, Chen L, Gu Y. Immune-defensive microspheres promote regeneration of the nucleus pulposus by targeted entrapment of the inflammatory cascade during intervertebral disc degeneration. Bioact Mater. 2024;37:132–52. 10.1016/j.bioactmat.2024.03.020. 38549774 10.1016/j.bioactmat.2024.03.020 PMC10972768

[8] Yang RZ, Xu WN, Zheng HL, Zheng XF, Li B, Jiang LS, Jiang SD. Involvement of oxidative stress-induced annulus fibrosus cell and nucleus pulposus cell ferroptosis in intervertebral disc degeneration pathogenesis. J Cell Physiol. 2021;236:2725–39. 10.1002/jcp.30039. 32892384 10.1002/jcp.30039 PMC7891651

[9] Yang X, Chen Y, Guo J, Li J, Zhang P, Yang H, Rong K, Zhou T, Fu J, Zhao J. Polydopamine nanoparticles targeting ferroptosis mitigate intervertebral disc degeneration via reactive oxygen species depletion, iron ions chelation, and GPX4 ubiquitination suppression. Adv Sci (Weinh). 2023;10:e2207216. 10.1002/advs.202207216. 36951540 10.1002/advs.202207216 PMC10161035

[10] Wu PH, Kim HS, Jang IT, Intervertebral Disc Diseases. PART 2: A review of the current diagnostic and treatment strategies for intervertebral disc disease. Int J Mol Sci. 2020;21:2135. 10.3390/ijms21062135. 32244936 10.3390/ijms21062135 PMC7139690

[11] Yang YH, Gu XP, Hu H, Hu B, Wan XL, Gu ZP, Zhong SJ. Ginsenoside Rg1 inhibits nucleus pulposus cell apoptosis, inflammation and extracellular matrix degradation via the YAP1/TAZ pathway in rats with intervertebral disc degeneration. J Orthop Surg Res. 2022;17:555. 10.1186/s13018-022-03443-4. 36539815 10.1186/s13018-022-03443-4 PMC9768949

[12] Yapici FI, Bebber CM, von Karstedt S. A guide to ferroptosis in cancer. Mol Oncol. 2024;18:1378–96. 10.1002/1878-0261.13649. 38590214 10.1002/1878-0261.13649 PMC11161738

[13] Xiang Z, Zhang P, Jia C, Xu R, Cao D, Xu Z, Lu T, Liu J, Wang X, Qiu C, Fu W, Li W, Cheng L, Yang Q, Feng S, Wang L, Zhao Y, Liu X. Piezo1 channel exaggerates ferroptosis of nucleus pulposus cells by mediating mechanical stress-induced iron influx. Bone Res. 2024;12:20. 10.1038/s41413-024-00317-9. 38553442 10.1038/s41413-024-00317-9 PMC10980708

[14] Zhang W, Liu Y, Liao Y, Zhu C, Zou Z. GPX4, ferroptosis, and diseases. Biomed Pharmacother. 2024;174:116512. 10.1016/j.biopha.2024.116512. 38574617 10.1016/j.biopha.2024.116512

[15] Lei G, Zhang Y, Koppula P, Liu X, Zhang J, Lin SH, Ajani JA, Xiao Q, Liao Z, Wang H, Gan B. The role of ferroptosis in ionizing radiation-induced cell death and tumor suppression. Cell Res. 2020;30:146–62. 10.1038/s41422-019-0263-3. 31949285 10.1038/s41422-019-0263-3 PMC7015061

[16] Liu H, Forouhar F, Lin AJ, Wang Q, Polychronidou V, Sonim RK, Xia X, Stockwell BR. Small-molecule allosteric inhibitors of GPX4. Cell Chem Biology. 2022;29:1680–e16939. 10.1016/j.chembiol.2022.11.003. 10.1016/j.chembiol.2022.11.003 PMC9772252 36423641

[17] Yu L, Hao YJ, Ren ZN, Zhu GD, Zhou WW, Lian X, Wu XJ. Ginsenoside Rg1 relieves rat intervertebral disc degeneration and inhibits IL-1β-induced nucleus pulposus cell apoptosis and inflammation via NF-κB signaling pathway. In vitro cellular & developmental biology. Animal. 2024;60:287–99. 10.1007/s11626-024-00883-6. 10.1007/s11626-024-00883-6 PMC11014818 38485818

[18] Yu L, Hao Y, Peng C, Zhang P, Zhu J, Cai Y, Zhu G. Effect of ginsenoside Rg1 on the intervertebral disc degeneration rats and the degenerative pulposus cells and its mechanism. Biomed Pharmacother. 2020;123:109738. 10.1016/j.biopha.2019.109738. 31951975 10.1016/j.biopha.2019.109738

[19] Alolga RN, Nuer-Allornuvor GF, Kuugbee ED, Yin X, Ma G. Ginsenoside Rg1 and the control of inflammation implications for the therapy of type 2 diabetes: A review of scientific findings and call for further research. Pharmacol Res. 2020;152:104630. 10.1016/j.phrs.2020.104630. 31911245 10.1016/j.phrs.2020.104630

[20] Sun Y, Yang Y, Liu S, Yang S, Chen C, Lin M, Zeng Q, Long J, Yao J, Yi F, Meng L, Ai Q, Chen N. New therapeutic approaches to and mechanisms of ginsenoside Rg1 against neurological diseases. Cells. 2022;11:2529. 10.3390/cells11162529. 36010610 10.3390/cells11162529 PMC9406801

[21] Kong L, Liu Y, Li J, Wang Y, Ji P, Shi Q, Han M, Xu H, Li W, Li W. Ginsenoside Rg1 alleviates chronic inflammation-induced neuronal ferroptosis and cognitive impairments via regulation of AIM2-Nrf2 signaling pathway. J Ethnopharmacol. 2024;330:118205. 10.1016/j.jep.2024.118205. 38641079 10.1016/j.jep.2024.118205

[22] Guo J, Wang R, Min F. Ginsenoside Rg1 ameliorates sepsis-induced acute kidney injury by inhibiting ferroptosis in renal tubular epithelial cells. J Leukoc Biol. 2022;112:1065–77. 10.1002/JLB.1A0422-211R. 35774015 10.1002/JLB.1A0422-211R

[23] Elmounedi N, Keskes H. Establishment of intervertebral disc degeneration models; A review of the currently used models. J Orthop. 2024;56:50–6. 10.1016/j.jor.2024.05.020. 38784950 10.1016/j.jor.2024.05.020 PMC11109335

[24] Zhang YY, Hu ZL, Qi YH, Li HY, Chang X, Gao XX, Liu CH, Li YY, Lou JH, Zhai Y, Li CQ. Pretreatment of nucleus pulposus mesenchymal stem cells with appropriate concentration of H2O2 enhances their ability to treat intervertebral disc degeneration. Stem Cell Res Ther. 2022;13:340. 10.1186/s13287-022-03031-7. 35883157 10.1186/s13287-022-03031-7 PMC9327256

[25] Li Z, Qu B, Wu X, Chen H, Wang J, Zhou L, Wu X, Zhang W. Methodology improvement for network Pharmacology to correct the deviation of deduced medicinal constituents and mechanism: Xian-Ling-Gu-Bao as an example. J Ethnopharmacol. 2022;289:115058. 10.1016/j.jep.2022.115058. 35114343 10.1016/j.jep.2022.115058

[26] Gao W, Wang X, Zhou Y, Wang X, Yu Y. Autophagy, ferroptosis, pyroptosis, and necroptosis in tumor immunotherapy. Signal Transduct Target Therapy. 2022;7:196. 10.1038/s41392-022-01046-3. 10.1038/s41392-022-01046-3 PMC9208265 35725836

[27] Yuan H, Pratte J, Giardina C. Ferroptosis and its potential as a therapeutic target. Biochem Pharmacol. 2021;186:114486. 10.1016/j.bcp.2021.114486. 33631189 10.1016/j.bcp.2021.114486

[28] Tang D, Chen X, Kang R, Kroemer G. Ferroptosis: molecular mechanisms and health implications. Cell Res. 2021;31:107–25. 10.1038/s41422-020-00441-1. 33268902 10.1038/s41422-020-00441-1 PMC8026611

[29] Liang T, Gao B, Zhou J, Qiu X, Qiu J, Chen T, Liang Y, Gao W, Qiu X, Lin Y. Constructing intervertebral disc degeneration animal model: A review of current models. Front Surg. 2023;9:1089244. 10.3389/fsurg.2022.1089244. 36969323 10.3389/fsurg.2022.1089244 PMC10036602

[30] Xu Y, Cai F, Zhou Y, Tang J, Mao J, Wang W, Li Z, Zhou L, Feng Y, Xi K, Gu Y. Chen, L. Magnetically attracting hydrogel reshapes iron metabolism for tissue repair. Sci Adv. 2024;10:eado7249. 10.1126/sciadv.ado7249. 39151007 10.1126/sciadv.ado7249 PMC11328908

[31] Cao G, Yang S, Cao J, Tan Z, Wu L, Dong F, Ding W, Zhang F. The role of oxidative stress in intervertebral disc degeneration. Oxidative Med Cell Longev. 2022;2022(2166817). 10.1155/2022/2166817. 10.1155/2022/2166817 PMC8769842 35069969

[32] Cheng H, Liu J, Zhang D, Wang J, Tan Y, Feng W, Peng C. Ginsenoside Rg1 alleviates acute ulcerative colitis by modulating gut microbiota and microbial Tryptophan metabolism. Front Immunol. 2022;13:817600. 10.3389/fimmu.2022.817600. 35655785 10.3389/fimmu.2022.817600 PMC9152015

[33] Zhang Z, Yang K, Mao R, Zhong D, Xu Z, Xu J, Xiong M. Ginsenoside Rg1 inhibits oxidative stress and inflammation in rats with spinal cord injury via Nrf2/HO-1 signaling pathway. NeuroReport. 2022;33:81–9. 10.1097/WNR.0000000000001757. 34954769 10.1097/WNR.0000000000001757

[34] Yuan K, Yang Y, Lin Y, Zhou F, Huang K, Yang S, Kong W, Li F, Kan T, Wang Y, Cheng C, Liang Y, Chang H, Huang J, Ao H, Yu Z, Li H, Liu Y, Tang T. Targeting Bacteria-Induced ferroptosis of bone marrow mesenchymal stem cells to promote the repair of infected bone defects. Adv Sci (Weinh). 2024;11:e2404453. 10.1002/advs.202404453. 39166412 10.1002/advs.202404453 PMC11497072

[35] Liu X, Wang W, Zhu F, Xu H, Ge G, Liang X, Yang H, Xu Y, Xu W, Wei M, Zhou Q, Geng D. Osteoblastic ferroptosis Inhibition by small-molecule promoting GPX4 activation for peri-prosthetic osteolysis therapy. J Nanobiotechnol. 2024;22:758. 10.1186/s12951-024-03049-4. 10.1186/s12951-024-03049-4 PMC11658433 39696565

[36] Ruan H, Zhu T, Wang T, Guo Y, Liu Y, Zheng J. Quercetin modulates ferroptosis via the SIRT1/Nrf-2/HO-1 pathway and attenuates cartilage destruction in an osteoarthritis rat model. Int J Mol Sci. 2024;25:7461. 10.3390/ijms25137461. 39000568 10.3390/ijms25137461 PMC11242395

[37] Huang L, Zhang S, Bian M, Xiang X, Xiao L, Wang J, Lu S, Chen W, Zhang C, Mo G, Jiang L, Li Y, Zhang J. Injectable, anti-collapse, adhesive, plastic and bioactive bone graft substitute promotes bone regeneration by moderating oxidative stress in osteoporotic bone defect. Acta Biomater. 2024;180:82–103. 10.1016/j.actbio.2024.04.016. 38621599 10.1016/j.actbio.2024.04.016

[38] Sun K, Shi Y, Yan C, Wang S, Han L, Li F, Xu X, Wang Y, Sun J, Kang Z, Shi J. Glycolysis-Derived lactate induces ACSL4 expression and lactylation to activate ferroptosis during intervertebral disc degeneration. Adv Sci (Weinh). 2025;12:e2416149. 10.1002/advs.202416149. 40171826 10.1002/advs.202416149 PMC12140309

