# Supplementary figures and images for: Does ginsenoside Rg1 promote intervertebral disc repair? An experimental study insights into ferroptosis mechanism
Source: J Transl Med. 2025 Nov 6;23:1231. doi: 10.1186/s12967-025-07047-4 (PMC12590719; doi:10.1186/s12967-025-07047-4)

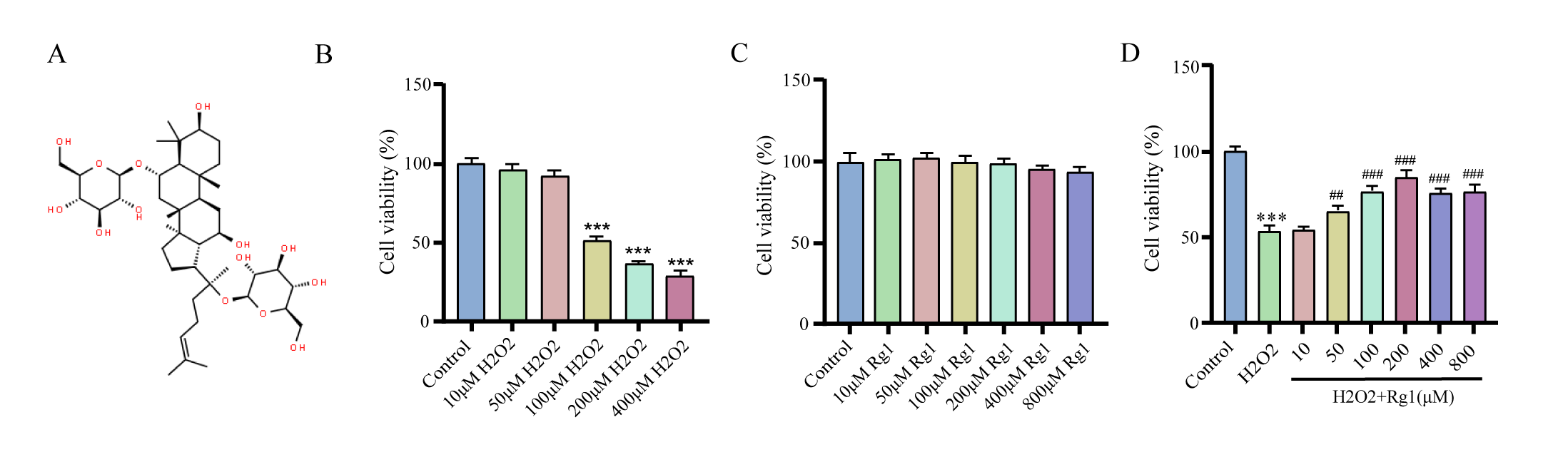

Supplement: Supplementary file 1 — Supplementary Material 1 [file 12967_2025_7047_MOESM1_ESM.tif]

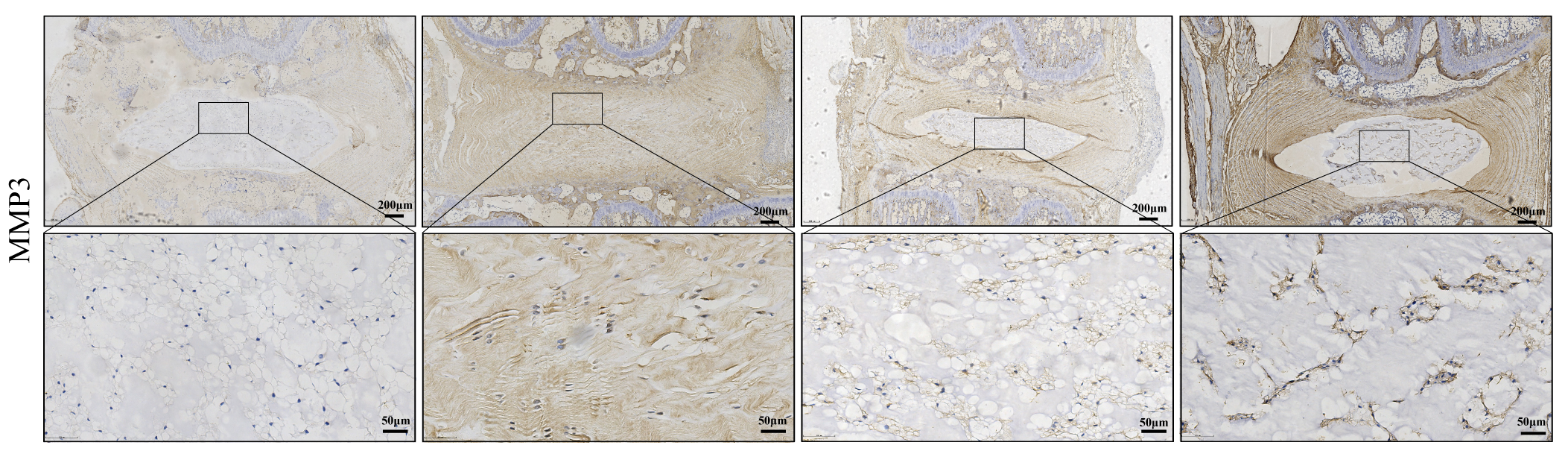

Supplement: Supplementary file 2 — Supplementary Material 2 [file 12967_2025_7047_MOESM2_ESM.tif]

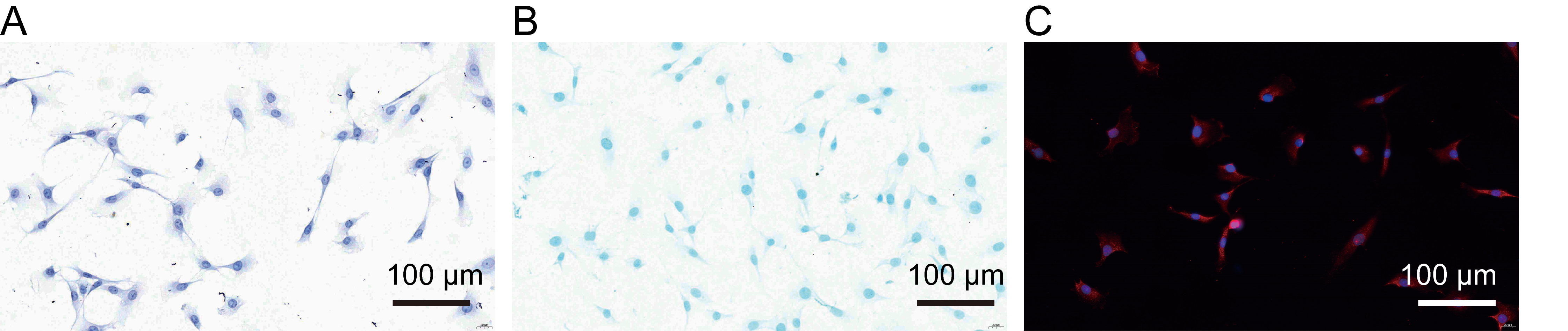

Supplement: Supplementary file 3 — Supplementary Material 3 [file 12967_2025_7047_MOESM3_ESM.tif]
