# Supplementary material for: Does ginsenoside Rg1 promote intervertebral disc repair? An experimental study insights into ferroptosis mechanism
Source: J Transl Med. 2025 Nov 6;23:1231. doi: 10.1186/s12967-025-07047-4 (PMC12590719; doi:10.1186/s12967-025-07047-4)
Supplement: Supplementary file 4 — Supplementary Material 4 [file 12967_2025_7047_MOESM4_ESM.docx]

**Human gene sequence RT-qPCR primer**

| **Gene** | **Primer** |
| --- | --- |
| GPX4 | Forward 5'-TCGGCCGCCTTTGCC-3'  Reverse 5'-TCCAGGTTAACCATGTGCCC-3' |
| FTL1 | Forward 5'-GCCACTTCTTCCGCGAATTG-3'  Reverse 5'-AGGGCCTGGTTCAGCTTTTT-3' |
| SLC7A11 | Forward 5'-GGTCAGAAAGCCTGTTGTGT-3'  Reverse 5'-TGCTCCAATGATGGTGCCAA-3' |
| GAPDH | Forward 5'-AATCCCATCACCATCTTC-3'  Reverse 5'-AGGCTGTTGTCATACTTC-3' |

**Rat gene sequence RT-qPCR primer**

| **Gene** | **Primer** |
| --- | --- |
| NFE2L2 | Forward 5'-GTTCCCCACTGCTCCGACTA-3'  Reverse 5'-TCTTGCCTCCAAAGGATGTCA-3' |
| GPX4 | Forward 5'-CCGTCTGAGCCGCTTATTGA-3'  Reverse 5'-CTGCGAATTCGTGCATGGAG-3' |
| SLC7A11 | Forward 5'-CGGGGTTGGCTTCCTTATCA-3'  Reverse 5'-GAGTCTTCTGGTACAACTTCTAGT-3' |
| FTL1 | Forward 5'-TCAAGTTGCAGAACGAACGC-3'  Reverse 5'-TCAGGTGGTTGCCCATCTTC-3' |
| GAPDH | Forward 5'-GGTGATGCTGGTGCTGAGTA-3'  Reverse 5'-TCACAAACATGGGGGCATCA-3' |
