# Supplementary material for: Does ginsenoside Rg1 promote intervertebral disc repair? An experimental study insights into ferroptosis mechanism
Source: J Transl Med. 2025 Nov 6;23:1231. doi: 10.1186/s12967-025-07047-4 (PMC12590719; doi:10.1186/s12967-025-07047-4)
Supplement: Supplementary file 5 — Supplementary Material 5 [file 12967_2025_7047_MOESM5_ESM.docx]

**Ginsenoside Rg1 molecular docking data**

| **Gene ID** | **PBD ID** | **Compounds** | **Estimated ΔG (kcal/mol)** | **Average** |
| --- | --- | --- | --- | --- |
| NRF2 | 2lz1 | ginsenoside Rg1 | -7.2 | 7.4 |
| NRF2 | 2lz1 | ginsenoside Rg1 | -7.6 |  |
| NRF2 | 2lz1 | ginsenoside Rg1 | -7.5 |  |
| GPX4 | 2obi | ginsenoside Rg1 | -5.5 | 5.6 |
| GPX4 | 2obi | ginsenoside Rg1 | -5.7 |  |
| GPX4 | 2obi | ginsenoside Rg1 | -5.8 |  |
| FTL1 | 3hng | ginsenoside Rg1 | -8.4 | 8.5 |
| FTL1 | 3hng | ginsenoside Rg1 | -8.7 |  |
| FTL1 | 3hng | ginsenoside Rg1 | -8.3 |  |
| SLC7A11 | 7ccs | ginsenoside Rg1 | -9 | 9.1 |
| SLC7A11 | 7ccs | ginsenoside Rg1 | -9.2 |  |
| SLC7A11 | 7ccs | ginsenoside Rg1 | -9.1 |  |
